# The elusive nature and diagnostics of misfolded Aβ oligomers

**DOI:** 10.3389/fchem.2015.00017

**Published:** 2015-03-19

**Authors:** Eleonora Cerasoli, Maxim G. Ryadnov, Brian M. Austen

**Affiliations:** ^1^Biotechnology Department, National Physical LaboratoryTeddington, UK; ^2^Basic Medical Sciences, St. George's University of LondonLondon, UK

**Keywords:** Aβ oligomers, neurodegeneration, protein misfolding, fibrillogenesis, Alzheimer's disease

## Abstract

Amyloid-beta (Aβ) peptide oligomers are believed to be the causative agents of Alzheimer's disease (AD). Though post-mortem examination shows that insoluble fibrils are deposited in the brains of AD patients in the form of intracellular (tangles) and extracellular (plaques) deposits, it has been observed that cognitive impairment is linked to synaptic dysfunction in the stages of the illness well before the appearance of these mature deposits. Increasing evidence suggests that the most toxic forms of Aβ are soluble low-oligomer ligands whose amounts better correlate with the extent of cognitive loss in patients than the amounts of fibrillar insoluble forms. Therefore, these ligands hold the key to a better understanding of AD prompting the search for clearer correlations between their structure and toxicity. The importance of such correlations and their diagnostic value for the early diagnosis of AD is discussed here with a particular emphasis on the transient nature and structural plasticity of misfolded Aβ oligomers.

## Protein conformational disorders

Cerebral proteopathies including Alzheimer's, Parkinson's, and Huntington's diseases result from progressive amyloidogenesis for which protein misfolding is the cause (Eisenberg and Jucker, 2012). The complexity of these diseases is the main reason behind the persistent lack of efficient therapeutic approaches. Traditional drug development relies on finding a therapeutic target which for most diseases can be traced down to an individual molecule, mutation or infection. In contrast, proteopathies are process disorders which develop over many years through a chain of conformational changes starting with an abnormally folded protein or/as in prion diseases, as a result of permissive templating of an endogenous protein by an exogenous amyloidogenic form. Therefore, proteopathies are often referred to as protein conformational disorders with a particular emphasis on a specific causative agent. This mini-review focuses on one of such agents—amyloid-beta or Aβ—and discusses the elusive nature of its development into cytotoxic oligomers (Meli et al., 2014).

**Figure 1:**
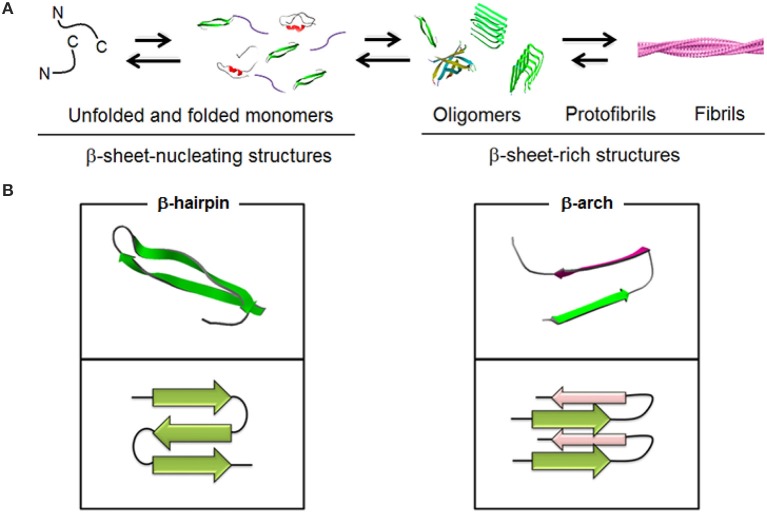
**Schematic representations of (A) spontaneous Aβ aggregation from β-sheet–nucleating monomers (lag) phase to β-sheet-rich oligomers, protofibrils and fibrils (PDB entries 1BEG, 2LFM, 2GSP, and 2OTK)**. Fibril model is reproduced under the terms of the creative Commons Attribution non-commercial license from Marshall (2009). **(B)** Two elementary units, β-hairpin and β-arch, leading to different assembly pathways (PDB entries 2OTK and 2LNQ rendered by Swiss PDB Viewer).

Aβ stands for a proteolytic product of a transmembrane amyloid precursor protein (APP). This is a 40–42 residue peptide which constitutes a primary structural component of insoluble fibrils accumulating in microscopic deposits, senile plaques, that are found in the post-mortem brains of Alzheimer's disease (AD) patients (Glenner and Wong, 1984; Masters et al., 1985). Early studies showed that in such plaques Aβ existed in a stable multimeric form and some credence was given to the role of Aβ in the disease by the demonstration that aggregated forms of Aβ were toxic to neuronal cells *in vitro* and *in vivo* (Walsh and Selkoe, 2007), whereas monomeric, soluble forms were innocuous. However, no correlation was found between the number of amyloid plaques in brain and the extent of cognition loss in examined brains of AD patients. Instead, better correlations were apparent between (i) the total amount of soluble and insoluble β-amyloid in brain and cognitive decline (McLean et al., 1999) and (ii) the amounts of Aβ oligomers and the extent of cognitive loss in patients as opposed to the number of fibrils (Glabe, 2008). These findings are in a striking agreement with that fibrils are less toxic to neurons than soluble oligomers (Sakono and Zako, 2010) and that lowering the cytotoxicity of β-amyloid does not necessarily reduce fiber formation (Zerovnik et al., 2011; Jiang et al., 2013). Therefore, Aβ fibrils can be viewed as repositories of soluble intermediates that are in equilibrium with insoluble forms (Bieschke et al., 2012) (Figure 1).

Outstanding questions relate to the precise relationships between the primary and secondary structure of Aβ and those tertiary interactions that underpin different polymorphic forms, transient or equilibrated, but which remain elusive to most experimental conditions that fail to adequately replicate Aβ aggregation pathways (Bitan et al., 2005; Philo, 2006). Yet again, it is becoming apparent that it is the conformational plasticity of Aβ which is responsible for the observed polymorphism and toxicity (Hubin et al., 2014). Along the same lines, it is reasonable to consider a sequence of nucleating processes each of which may potentially lead to different cytotoxicity (Necula et al., 2007; Miller et al., 2010). Such a notion prompts an important conclusion that key factors for the cytotoxic effects of β-amyloid may be confined to interactions between soluble oligomers and cellular membranes (Broersen et al., 2010), (Broersen et al., 2010; Stefani, 2012) favoring stronger binders (Miller et al., 2010).

## Aβ oligomerisation: conformation, size and elementary units

In fibrils Aβ is arranged into a parallel, in-register cross-beta architecture in which individual β-sheets run perpendicular to the fibril axis. A β-turn-β arc, which in contrast to a β-hairpin is side-chain-bonded, is reported as an elementary unit for both fibrils and nucleators (Kajava et al., 2010) but remains unrecognized as a dominant conformation for oligomers. Largely this is due to the more pronounced conformational plasticity of Aβ in low oligomeric structures which are prone to specific changes in response to environmental factors (Miller et al., 2010).

Encouragingly, such a permissive conformational background as well as polymorphic plasticity allow for the development of conformation-dependent oligomer-specific antibodies. For example, OC polyclonal antibodies recognize fibrils or fibrillar oligomers but not pre-fibrillar oligomers which though have broadly the same sizes are immunologically different and are recognized by A11 antibodies (Kayed et al., 2003, 2007). Fibrils and pre-fibrillar oligomers therefore have different conformation-dependent epitopes which suggests that oligomer conformations are likely to be main determinants of cytotoxicity (Ladiwala et al., 2012). Although pre-fibrillar oligomers can vary in size and morphology (Benilova et al., 2012) their variety is dependent on experimental conditions used (Lee et al., 2007; Gillam and MacPhee, 2013). The size alone thus cannot serve as a reliable indicator of oligomer conformation or toxicity. Similarly, the morphology of soluble aggregates, which tends to be dominated by spheroidal forms (Lambert et al., 1998), seem to bear little relevance to size-dependent toxicity. Low oligomers, often termed amyloid-derived diffusible ligands (ADDLs), are believed to range in diameter from 1 to 15 nm (Hoshi et al., 2003), with similar morphologies expressed at the micrometer scale (Westlind-Danielsson and Arnerup, 2001). β-hairpins stabilized into U-shape conformations are maintained in these structures. Deviations in in-register β-sheet arrangements occur with increasing size when the assembly is forced to adopt a twist which destabilizes further fibril-like growth thus limiting the assembly to lower oligomers. In contrast, twist pairs as small as Aβ pentamers can be stabilized through a hydrophobic interface to seed conformational templates for fibrillogenesis (Kahler et al., 2013).

However, any assignment of a specific conformation is hampered by the heterogeneity and instability of lower oligomers, and consequently questions the existence of a specific toxic oligomer (Hubin et al., 2014). Analysing the dynamics of the conformational space available to Aβ may provide better insights. For example, proteolytic cleavage of SDS-stable globule-like aggregates helped to reveal a C-terminal part of Aβ sequence buried in the hydrophobic core suggesting conformational adaptability of a solvent-exposed N-terminal fragment (Barghorn et al., 2005). Proposed as intermediates toward fibril formation (Chimon and Ishii, 2005) these structures were found to be toxic pre-fibrillar oligomers (A11-positive) suggesting that the supramolecular packing of a similar elementary β-sheet unit is different from that in fibrils (Chimon et al., 2007). In pre-fibrillar oligomers the unit was found to be an anti-parallel β-sheet followed by an in-register, parallel intermolecular sheet (Olejniczak et al., 2008; Yu et al., 2009). With the dimer covalently locked through an introduced disulphide bond, the oligomers could still be recognized by the same antibody suggesting constraint-independent binding (Yu et al., 2009). More recent evidence broadens potential pathways to toxic oligomers with mechanisms involving out-of-register β-sheets (Liu et al., 2012) and staggered anti-parallel arrangements (Tay et al., 2013).

In this light, revealing an elementary monomeric conformation appears to be timely and appealing. In an exemplary attempt, an Aβ monomer covalently locked into a stabilized β-hairpin was shown to associate into oligomeric and pre-fibrillar structures but not mature fibrils, while, remarkably, exhibiting cytotoxicity and antibody recognition typical of native oligomers (Sandberg et al., 2010; Dubnovitsky et al., 2013). These results confirm that Aβ peptide (primary structure) folded into a hairpin (secondary structure) constitutes the elementary monomeric motif supporting the formation of oligomers.

However, links between the elementary units and their tertiary arrangements in oligomers are far less clear. Some evidence points in favor of oligomeric intermediates having specific structural topologies, e.g., assembled β–barrels, which are A11-positive. Given that the binding of A11 is sequence-independent and that it recognizes other non-amyloid oligomers including α-haemolysin and heat shock proteins (Yoshiike et al., 2008), these proposed topologies are likely to be off-pathway intermediates. For the same reason, the structures could represent oligomeric forms adopted by other amyloid-forming peptides, and can be used as β-sheet amyloid mimetics.

Uncertainties in providing explicit relationships between secondary and tertiary structures in transient oligomers encourage alternative representations of monomeric units. One of such alternatives is an atypical secondary structure motif—an α-sheet. Similar to β-sheets this structure forms pleated structures which however differ by that all peptide-bond carbonyls lie in the same direction on one side of the pleat while all amino groups mirror the arrangement on the opposite side of the pleat. This enables opposite charges on the opposite edges of one pleat, also referred to as a polar pleated sheet, and more pronounced sheet flattening (Pauling and Corey, 1951; Armen et al., 2004). Peptides designed to adopt this secondary structure were able to bind toxic oligomers of two unrelated amyloid proteins, Aβ (1-42) and transthyretin (TTR) (Armen et al., 2004; Hopping et al., 2014). The structure is also considered as promising for addressing the main paradox of structure-activity relationships in amyloidogenesis by delineating the dependence of the process on peptide sequences from that it is mainly driven by backbone stabilization factors.

Yet, this and any other alternative structures can only provide indirect evidence which would require validation for native amyloidogenic sequences.

## Aβ oligomers: toxicity and synaptic degeneration

AD is the most common form of dementia which affects episodic memory. Plasticity of the synapse would appear to be intimately involved (Martin et al., 2000), whereas the structure of the synaptic network changes in response to external or internal stimuli enabling at a later date the repeated access to those stimuli. Thus, the most damaged facet of memory in AD is the loss of semantic memory, the store of factual and conceptual knowledge that is not linked to a specific memory.

Changing the strength of synaptic connections between neurons is widely thought to be the mechanism by which memory traces are encoded and stored in the brain. This hypothesis states that activity dependant synaptic plasticity is induced at appropriate synapses during memory formation and is necessary and sufficient for the information storage underlying memory. An exemplary insight into how Aβ production and aggregation may give rise to memory loss *in vivo* (Kayed and Lasagna-Reeves, 2013) can be demonstrated by a recent finding (Russell et al., 2012).

Specifically, Aβ oligomers blocked long-term potentiation (LTP) in the hippocampal slices of fetal rats. LTP is an experimental paradigm of synaptic plasticity, which is characterized by an increasing response with repeated stimulation over time. Aβ oligomers at nanomolar levels derived by incubation of synthetic Aβ, or isolation from cellular supernatants, blocked maintenance but not initiation of LTP. Blockage was alleviated by the additional presence of protecting antibody or the application of scrambled Aβ (Walsh et al., 2002). There is plenty of direct experimental evidence to support the notion of toxic oligomers (Walsh et al., 2002; Kayed et al., 2003, 2007; Haass and Selkoe, 2007; Tomic et al., 2009; Mucke and Selkoe, 2012; Lesne et al., 2013), also suggesting that the activity of causing dementia may occur across a broad oligomer range. For example, Aβ dodecamers were identified in human brain extracts and found to bind cultured neurons in a manner similar to synthetic ADDLs (Lacor et al., 2004). The implication of these brain-derived dodecamers as pathophysiologically relevant Aβ oligomers is supported by the isolation of a similar 56 kDa oligomer from APP-overexpressing AD transgenic mice that was capable of disrupting memory upon injection into young wild type rats (Lesne et al., 2006) At the lower end of the oligomer spectrum a particular neurotoxic effect is assigned to dimers and trimers (Hung et al., 2008; Ono et al., 2009), which were also found to inhibit LTP in hippocampal slices (Shankar et al., 2007, 2008).

Amyloid oligomers block LTP in hippocampal neurones by binding and cross-linking receptors that are then internalized and degraded in the post-synaptic neuron. Drastic neuronal damage also occurs. Loss of neuronal density and synapse concentration is a marked characteristic of a post-mortem AD brain, and cognition loss can occur when levels of soluble oligomers are high, many months before the appearance of senile plaques (Moechars et al., 1999). Low concentrations (pM) of β-amyloid, commensurate with levels found in normal human brain, produced a marked increase in LTP in hippocampal slices (Puzzo et al., 2008), whereas larger concentrations (nM) led to a block raising the possibility that the normal function of Aβ may be to modulate synaptic plasticity positively. This finding raises the problem that any therapeutic reduction in Aβ may have to leave trace amounts of Aβ to function during normal development. Indeed, soluble oligomeric forms in aqueous extracts of post-mortem brains from patients showing early cognitive loss contained SDS-stable oligomers suggesting their early accumulation in the disease process (McLean et al., 1999).

**Figure 2:**
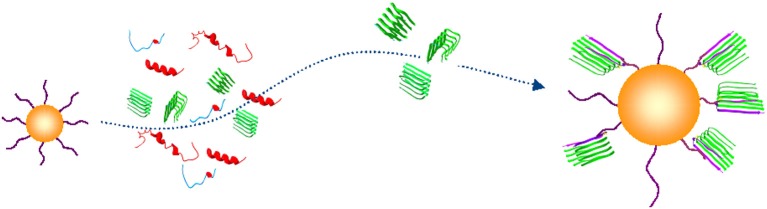
**Conceptual schematic of the nanoprobe-based capture of Aβ oligomers**. A capturing bait conjugated to a gold nanoparticle (yellow) discriminates an Aβ oligomer in β-sheet conformation (green) from unfolded or folded monomers (blue and red) (PDB entries 1BEG, 2LFM, 2GSP, and 2OTK). (Georganopoulou et al., 2005; Chikae et al., 2008; Santos et al., 2008; Herskovits et al., 2013).

Direct implications of these and other findings is the necessity of diagnostic detection platforms that would be able to capture and detect circulating trace amounts of Aβ oligomers (Figure 2).

## Final remarks on the diagnostic value of Aβ

Despite the need and urgency and that all experimental evidence points to the substantial value of Aβ oligomers for early diagnosis and as a possible surrogate biomarker in clinical trials (Sian et al., 2000; Austen et al., 2008; Gao et al., 2010; Yam et al., 2011; Herskovits et al., 2013; Lesne et al., 2013), Aβ has yet to be validated as a clinically accepted marker for AD (Hampel et al., 2010; Humpel, 2011; Chintamaneni and Bhaskar, 2012; Rosen et al., 2013).

Admittedly, the lack of progress in this area is due to the outlined uncertainties of amyloid formation, which compromise the very notion of efficient diagnosis as a synergistic balance between specificity and the speed of obtaining results. However, given that the post-mortem analysis of *ex-vivo* brain specimens remains the only definitive AD diagnosis any test able to differentiate soluble oligomers at an early pre-fibrillar stage is of significant value and must be tried.

The development of technologies based on conformation-responsive detection of Aβ is arguably the most appealing approach toward this. The diagnostic value of such a strategy is based on direct correlations between oligomer amounts and disease progression, but yet again stumbles upon our poor understanding of precise conformational states that are responsible for AD. Revealing these conformations, transient or stable, may underpin the development of new molecular modalities with differentially selective and high affinity profiles to soluble oligomers that can be used as molecular sensors or baits enabling the capture of amyloid precursors in biological matrices.

### Conflict of interest statement

The authors declare that the research was conducted in the absence of any commercial or financial relationships that could be construed as a potential conflict of interest.

## Abbreviations

AD: Alzheimer's disease
APP: amyloid precursor protein
ADDLs: amyloid-derived diffusible ligands
TTR: transthyretin
SDS: sodium dodecyl sulphate.
